# Understanding young adult Muslim and Christian attitudes toward queer identity: A Canadian and UK comparison

**DOI:** 10.1177/1363460721995756

**Published:** 2021-02-21

**Authors:** Sarah-Jane Page, Heather Shipley

**Affiliations:** Aston University, UK; 6363 University of Ottawa, Canada

**Keywords:** Sexuality attitudes, queer, embodiment, youth, religion, Christian, Muslim

## Abstract

This article utilises data from two parallel mixed-methods projects to examine attitudes to queer identity among heterosexual Muslim and Christian young adults aged between 18 and 25 in Canada and the UK. Drawing on questionnaires, interviews and video diaries, our analysis revealed the enormous complexity regarding how participants made sense of their, largely contextually mediated, attitudes. A qualitative approach enabled us to carefully consider how these attitudes are forged, formulated and embodied, offering greater nuance and detail compared with attitudinal approaches focused solely on quantitative data. Responses by these young Muslim and Christian participants explore the fluidity and movement in the attitudes expressed, further emphasising complexity.

## Introduction

This article focuses on how attitudes to queer identity by heterosexually identifying religious young people were formulated in Canada and the UK. We define queer as those identifying with an array of sexual identities beyond heterosexual, including those who choose to not label their sexuality. We start by discussing the contextual and relational nature of attitude formation, and how they are rooted in societally based emotional investments. Although homophobia has typically been understood as a fear-based psychological reaction to queer identity, we argue a more nuanced and complex understanding of emotion and attitudes is needed. We turn next to our methodological approach, given that the data draw from two different studies. In order to tease out the complex ways attitudes are forged, we argue that a mixed methods approach is best, drawing on quantitative and qualitative means. We will outline the ‘tick box’ findings to the questionnaires, indicating that Muslim and Christian heterosexual young people were far more likely to be opposed to queer identity. Given their higher likelihood to have a conflicted or oppositional stance, we further explore the attitudes of Christians and Muslims in greater detail, through examining the qualitative data. We consider how those opposed to queer identity did their utmost to present good personhood, distancing themselves from any notion of fear of queer people, utilising instead emotions such as love. Next we examine the individuals who took an egalitarian view, unsupported by their religious tradition, examining how this was emotionally managed. We then consider those individuals whose attitude changed over time, prompting questions regarding how significant queer issues were for heterosexual participants overall. Finally, the discussion and conclusion section outlines some implications regarding religious attitudes.

### Mapping attitudes

Blumer’s (1936) classic work on the formulation of attitudes points to their cultivation in a social environment. Attitudes do not emerge from nowhere, but are contextually determined through interaction and interpretation. Blumer details two stages in the composition of attitudes. The *symbolic aspect* is how we verbalise and rationalise our views to ourselves and others; undertaken primarily by interpretive work. The *affective aspect* is the immediate judgements we make, capturing our emotional and non-verbal responses. These predispositions are not biologically driven, nor are they individualised. They are cultivated through interaction with others, taking on collective sentiments, even as they change over time. In Blumer’s account, the symbolic and affective imply a separation of emotional and rational processes. Meanwhile for Midgley (2004), the emotional and the rational are embedded in each other. Feelings involve thought, and thoughts involve feelings. Our judgements regarding the ‘wrongness’ of something – e.g. slavery or animal cruelty – is usually accompanied by strong feelings. Both theorists emphasise that attitudes are not solely about so-called rational processes, but are embodied. Take the example of food tastes and disgust. Rhys-Taylor (2013) analyses the reaction of a mother and daughter eating jellied eels. Whereas the elderly mother, a long-standing resident of London’s East End, eats the jellied eels enthusiastically, her daughter, a professional woman, looks on in disgust: ‘the nose, taste buds and stomach combined are … guardians of generational senses of difference’ (Rhys-Taylor, 2013: 241).

Arguably, this visceral response does not constitute an attitude in and of itself. As Voas (2013) emphasises, for something to be considered an attitude, it has to be directed beyond the individual, formulating an articulation of how society *should* operate (e.g. opposition to slavery). In Voas’s argument, if an individual prefers liquorice over chocolate, this is a personal preference, having few social consequences – a person with an aversion to chocolate is not asking that all people refrain from eating chocolate. Still, Blumer’s argument is that these issues are interconnected; these emotional responses can develop to produce a social effect akin to an attitude. Returning to the jellied eels – Rhys-Taylor’s argument is not simply that these negative reactions form a like or a dislike; they actually constitute a *classed* response to particular working-class behaviours. The socially mobile daughter, having cultivated a contrastive identity to her working class roots, sees the jellied eels as distasteful. This distaste mimics the broader cultural positioning of working-class individuals not only as different, but as *other*, these taste classifications are utilised to situate working class individuals as inferior (Tyler, 2013). As Ahmed (2004) argues, objects deemed disgusting come to represent borders between individuals and groups. In this case, the jellied eels represent class disgust. Through taste preferences, attitudes are forged. Our embodied responses connect to how we rationalise, interpret and generate attitudes about the world. And these responses are contextually mediated.

Ahmed (2004) states that emotion should be understood in terms of cognition and bodily expression, affirming the idea that emotions are a form of embodied judgement. For her, there is a relational element to emotions; our reactions to people and objects are subject to various histories – both personal and collective – determining whether we see something in beneficial or harmful terms. This allows for much variance in interpretation. Our feelings toward something or someone are not inherent in that person or object, but are ‘produced as effects of circulation’ (2004:8) – the eels are not inherently disgusting; the feeling generated is instead dependent upon the relationship cultivated between the individual and the eels.

This can be mapped onto responses to queer identity. As Fone explains, negativity to homosexuality is ‘founded upon fear and dislike of the sexual *difference* that homosexual individuals allegedly embody’ (2000:5, emphasis in original). This comprises an emotional, visceral reaction – relating not only to individual choices regarding one’s own sexual preferences, but having broader consequences for how one imagines the world should be. In other words, it is often assumed that being opposed to queer identity indicates an individual who is not only making a choice about oneself (to *not* be queer) but is articulating an ideal society being one where queer identities are non-existent. Bodily discipline has been crucial to this. Historically in England and Canada, the ‘sodomite’ has been varyingly punished through execution, life imprisonment and hard labour (Corriveau, 2011; Fone, 2000). Medicalised disciplinary regimes have encouraged hormone ‘therapies’ to suppress sexual inclinations. The body is therefore centrally positioned for discipline (Foucault, 1978). Opposition has historically not stopped at mere dislike of queer sexual behaviour but has cultivated explicit censure and control, expressing not only disgust, but punitive exclusion. Therefore, attitudes to queer people have – in both historic and contemporary environments – been embodied. Homosexuality is no longer criminalised in the UK or Canada; legislation in both countries now supports sexual equality. However, cultural legacies have ongoing impact (Ahmed, 2004).

When making sense of negative reactions to homosexuality, the notion of homophobia is often deployed. As Kulick explains, its rootedness in a psychological fear of homosexuality ‘locate(s) the source of prejudice against homosexuals not in social structures but in the individual psyche’ (2009: 23). This prioritises an individual pathology that downplays the social context for such a response, with homophobia lacking an alignment with terms such as sexism, and racism, where the terms themselves indicate societal-level causes. But Kulick’s point is that although it is problematic for terms such as homophobia to evade the social, it is equally problematic for terms like sexism and racism to avoid reference to the emotion-work that is undertaken by individuals in order for those structures to have salience. Although homophobia is a problematic concept for viewing negativity to homosexuality one-dimensionally (in terms of fear) and being primarily concerned with psychological responses, it does at least alert us to the importance of focusing on the kinds of emotion invested in attitudes towards homosexuality – inevitably going beyond fear. This focus on emotionality enables greater nuance in understanding attitudes, and the probable consequences. Is the level of emotion, positive or negative, invested in the issue likely to lead to a queer person being: Tolerated? Ignored? Verbally insulted? Will their very body be at risk of violence? As Murray argues, ‘discrimination against homosexuals can be conveyed through a range of attitudes: from indifference to dismissal, “scientific” logic, “tolerance,” or even a carefully delimited embrace (as in “love the sinner, not the sin”)’ (2009: 3). In recent years, the concept of heteronormativity has been utilised in order to understand the positioning of queer sexuality in contexts of greater acceptance (Richardson, 2018). Although it may be the case that legal provisions and broader attitudes have become more welcoming, this does not necessarily mean that queer sexualities are deemed equal, so that treating heterosexuality as the norm can still be a dominant feature of societies which on the surface promote queer equality (Jackson, 2006; Page and Shipley, 2020).

### Queer identity in the UK and Canada

In 1988, Section 28 prohibited the promotion of homosexuality in schools across England, Wales and Scotland (later repealed). In 1995, the *Canadian Charter of Rights and Freedoms* (1982) had its equality rights (section 15) provision challenged as it did not include sexual orientation as a protected right (*Egan*). More recently, there have been legal shifts endorsing sexual equality. The *Civil Partnership Act 2004* recognised the relationships of same-sex couples, further enhanced through the legalisation of same-sex marriage (England and Wales 2013; 2014 in Scotland and 2020 in Northern Ireland). In 1998, the Supreme Court of Canada ordered that sexual orientation be added to all provincial human rights codes as a protected ground (*Vriend*). In 2003, Ontario was the first province to recognise same-sex civil unions (*Halpern*), resulting in additional provincial changes and culminating in the *Reference Re Same-Sex Marriage* in 2004 and *The Civil Marriage Act* in 2005.

The UK and Canada are understood as liberal democratic societies with constitutional and human rights expectations, although rights for queer identities have only been embedded relatively recently. Sexual attitudes are liberalising, particularly amongst young people (Adam and Maticka-Tyndale, 2011; Maticka-Tyndale, 2008; Weeks, 2007). There is increasing support for same-sex relationships (Hooghe and Meeusen, 2013; Ross and Sacker, 2010; Yip, 2012; Young, 2012). But correlations have been noted between religious identification and conservative views to sexual diversity, particularly individuals following Abrahamic traditions (e.g. Clements, 2015; Roggemans et al., 2015; Rosik et al., 2007; Yip, 2012). In Western democratic societies where support for queer rights has been forged, it is typically religious identity, rather than other factors, which determine a conservative attitude to queer individuals (Adamczyk, 2017). Comparing global attitudes to homosexuality on the basis of religion, Adamczyk (2017) concludes that conservative Protestants, as well as Muslims, are the two religious groups most likely to condemn homosexuality, rooted in traditional interpretations of sacred texts (see also Roggemans et al., 2015). In the British context, Siraj (2009) notes an alignment between conservative religious interpretations and negative attitudes amongst Muslims. Her participants ‘did not perceive being homosexual as a legitimate social, personal or religious identity’ (2009: 55). The Roman Catholic Church, too, has been profoundly negative towards queer communities (Petro, 2015; Wilcox, 2018), but Adamczyk (2017) argues that individual Catholics are likely to take a more liberal view. Therefore, the relationship between religious pronouncements and individual attitudes is not clear-cut. Woodhead’s (2013) British survey of religious individuals identified 8.5% constituting a conservative ‘moral minority’ on sexuality matters, likely to emerge from conservative Christian traditions and Islam, often endorsing conservative teaching. Meanwhile, other religiously identifying individuals not constituting the moral minority (the vast majority) are more likely to forge their views on personal life on their own terms:

Overall, it is interesting to see how little our views on personal morality seem to be shaped by factors such as gender, religion, class, ethnicity, education and political views … . The only thing which has a striking effect on ethical views is being strictly religious. (Woodhead, 2013:8)

For Woodhead, it is the strength of religiosity and close alignment to traditional interpretations of religious pronouncements on sexuality issues, which makes a difference. This article concentrates mainly on young adult heterosexuals emerging from conservative traditions within Christianity and Islam, to understand how they themselves interpret conservative teaching towards homosexuality, and the extent to which they emotionally invest in conservative pronouncements.

## Methods

We present findings from two projects from the UK and Canada, which mirrored each other (Page and Shipley, 2020; Yip and Page, 2013; Young and Shipley, 2020). The UK project focused on how religious young adults (aged 18–25) navigated sexuality, incorporating people of varying sexual orientations. The Canadian project^1^ was identical, although it also included those who did not identify religiously. For the purposes of this article, the focus is religious individuals. Both projects included questionnaires, in-depth interviews and video diaries. Many of the questions were either identical or very similar; revisions in the Canadian study included additional questions about same-sex marriage, which had not been legalised in the UK at the time of data collection, and the expansion of the religious identity categories.^2^ Each project was conducted by different research teams, in the UK (2009–2011) and Canada (2012–2016).

Each sample was similarly devised, with recruitment among youth-based secular and religious organisations. This created a diverse sample comprising a range of religious traditions, genders and sexual orientations. Both projects were based on a purposive heterogeneous sample that was not representative of the broader population (Spencer and Pahl, 2006); we are not making claims about the strength of the statistics beyond our own datasets. Nevertheless, these studies are significant in mapping how the same individuals responded to questions about queer identity across three methods, giving rich and compelling results.

This article focuses on those identifying as both religious and heterosexual. Within the UK sample, 515 individuals completed the questionnaire, 34 were interviewed and 13 completed a video diary. The religious traditions included Christian (57.9%), Muslim (18.8%), Hindu (8%), Jewish (6.2%), Sikh (3.7%), mixed faith (comprised of those who belonged to multiple religions – 3.1%) and Buddhist (2.3%). Regarding gender, 69.5% were women. In the Canadian sample, 215 individuals identified as religious and heterosexual, 21 of whom were interviewed. These participants identified as heterosexual, though some added to that identity ‘a little flamboyant,’ ‘mostly straight’ and ‘to the best of my knowledge’. The overall sample from the Canadian project identified as follows: Buddhist (1.4%), Christian (85.3%), Hindu (1.9%), Muslim (6.6%), Jewish (3.8%), Sikh (0.5%) and Spiritual-but-not-religious (14.7%).^3^ Of those identified as heterosexual, 73.1% identified as female, although some complicated that category (e.g. gender fluid), 26.6% identified as male, and 0.4% identified as trans.

Mapping attitudes to queer identity featured at various stages. The questionnaire contained three attitudinal statements about their respective attitudes, which elicited a Likert-scale response (from strongly agree to strongly disagree). The three statements were: ‘Heterosexuality should be the only expression of human sexuality’; ‘Heterosexuality is the ideal for human sexuality’; ‘Heterosexuality and homosexuality should be treated equally’. The first statement offers a non-negotiable stance, with heterosexuality deemed as the only accepted form of sexual expression. The second statement privileges heterosexuality but does not necessarily exclude other sexual expressions. Both of these statements are framed as heteronormative and assume the normativity of heterosexuality. The third statement elicited an equality position. Questionnaire responses were clarified within the interviews. For the video diary, participants recorded themselves everyday for a week, reflecting on sexuality and religion issues without researcher intervention. This highlighted issues that emerged in their everyday lives, e.g. books they were reading (Page, 2017; Yip and Page, 2013). We will start with assessing the quantitative data of the whole sample (covering all religious categories), then moving onto the qualitative data, where we solely consider Christian and Muslim participants, who were typically managing conservative teachings regarding homosexuality within their religious tradition.

### Tick box attitudes

Within the UK study, 39.2% of heterosexual participants strongly agreed or agreed with the first questionnaire statement that ‘Heterosexuality should be the only expression of human sexuality’. The comparable figure for Canada was 20%. For the second statement, ‘Heterosexuality is the ideal for human sexuality’, 61.8% of the UK sample strongly agreed or agreed, compared with 32.1% in Canada. The final statement, ‘Homosexuality and heterosexuality should be treated equally’, was supported by 51.3% of UK participants, compared with 75.6% for the Canadian sample.^4^

Canadian responses were more liberal; fewer identified with the most conservative statement, and more endorsed an equality position. This could be attributed to the greater embedding of equality rights in Canada, with marriage equality a reality at the federal level since 2005. Canadian participants experienced these equality rights since they were anywhere as young as eight years old. Meanwhile, some of the older UK participants still remembered experiencing Section 28 during their education. Further, the differing time frames as to when the projects were conducted could impact the ways participants responded to these questions. Significant social changes have occurred between the UK capture (2009–2011) and the Canadian capture (2012–2016) which could also influence how participants viewed sexual identity(ies).

Religious identification had an impact regarding how attitudes were formulated at the questionnaire stage. Tables 1 [Table table2-1363460721995756]to 3 detail the differences between religious traditions. In the UK, Muslim participants are far more conservative in their response, followed by Christian participants. In Canada, Christian (evangelical and Roman Catholic) and Muslim respondents were less supportive of sexual diversity. In both countries, Christians and Muslims are more conservative.

**Table 1.** Heterosexual participants who ‘strongly agreed’ or ‘agreed’ with ‘Heterosexuality should be the only expression of human sexuality’.

Religious identification	UK Percentage	Canada percentage
Buddhist	8.3	N/A
Christian	41.5	29.6
Hindu	9.8	N/A
Jewish	6.5	16.7
Mixed faith (UK)	18.8	N/A
Muslim	70.0	23
Sikh^6^	11.1	N/A
SBNR (Can)	N/A	N/A
No. of valid cases	497	212

**Table 2. table2-1363460721995756:** Heterosexual participants who ‘strongly agreed’ or ‘agreed’ with ‘Heterosexuality is the ideal for human sexuality’.

Religious identification	UK percentage	Canada percentage
Buddhist	33.3	N/A
Christian	66.0	44.4
Hindu	35.9	N/A
Jewish	24.1	16.7
Mixed faith (UK)	46.7	N/A
Muslim	84.4	46.2
Sikh	31.6	N/A
SBNR (Can)	N/A	9.7
No. of valid cases	495	211

**Table 3.** Heterosexual participants who ‘strongly agreed’ or ‘agreed’ with ‘Heterosexuality and homosexuality should be treated equally’.

Religious identification	UK percentage	Canada percentage
Buddhist	83.3	100
Christian	47.8	79.3
Hindu	80.0	66.7
Jewish	77.4	100
Mixed faith	62.5	N/A
Muslim	28.6	31
Sikh	83.3	N/A
SBNR (Can)	N/A	100
No. of valid cases	497	210

These patterns reaffirm the findings of other studies highlighting religious conservatism. We will now assess the qualitative methods, revealing greater complexity.

### Narrating good personhood

In-depth interviews generated extensive justifications for participants’ attitudes. Those opposed to queer identities knew this was an unpopular viewpoint more broadly and defended their position to emphasise ‘good personhood’. This was often achieved through separating action and person, so they could unequivocally claim that they held no animosity:

I have nothing against the people but I don’t think it is right to be gay or lesbian. (Louise, heterosexual non-denominational Christian woman, UK)

[I]f I … gave my articulation in ten minutes or less of sexual morality I would be labelled a homophobe … .what I’ve said deserves a fair hearing and it should be treated with respect … I just have some sort of rational reasons for [my attitude]. (Anthony, heterosexual Roman Catholic male, Canada)

A lot of people think Catholics hate homosexuals, which is entirely not true. Like there’s always the phrase, “hate the sin, not the sinner”. Yes, Catholic teaching … believes that homosexual acts are wrong … there is also some scientific evidence to show that … say homosexual - families that have two parents of the same sex aren’t necessarily as productive as a traditional family … so it’s important to … love everyone including homosexuals … it’s not necessarily their fault that they feel the way they do. They are not bad people (Jasmine, heterosexual Roman Catholic woman, Canada)

These participants distanced themselves from negative terminology such as ‘homophobia’ rejecting any suggestion that they disliked queer people. If participants disclosed an emotive reaction, this was conveyed positively as the ‘love’ they felt, whatever someone’s sexual orientation. However, this was often disingenuous as it was typically managed through separation of persons and acts, with a number of accounts mentioning the mantra, ‘love the sinner, hate the sin’ (Jakobsen and Pellegrini, 2003; Moon, 2004). Underscored by Christian language that they felt was inclusive, this still positioned queer people as sinful. Ahmed (2004) argues that emotion and sensation are hard to divide; love is the corollary of pleasure and hate is the corollary of pain. Therefore, one who feels the sensation of pleasure would correspondingly evoke an emotion of love. Yet here, participants articulate disconnect between the emotion and sensation; love is accompanied not by pleasure, but by pain or anguish or simply the feeling that they were right. Therefore, at the heart of their expression is a fundamental contradiction; they make sincere verbal attempts to accommodate queer people, while at the same time not following this through at the level of feeling.

Participants often foregrounded scientific narratives as justification. Referencing science gave their attitude greater credibility in an environment where scientific discourses are taken more seriously than religious ones. Participants did not reveal negative visceral responses, and instead crafted their justification to oppose queer identity in seemingly rational terms. Their attitudes were cultivated in a broader environment of acceptance of sexual diversity; they knew their viewpoint was an unpopular one. Those opposing queer identity therefore utilise positive emotional responses to evoke good personhood (which is put into doubt when opposition to queer identity is disclosed).

### Egalitarian compromises

Participants professing an equality position highlighted the problems they encountered from their religious organisation to which they belonged, which was not always as inclusive as they were:

[T]hey view them as kind of ‘Like look how great we are because we accept them’, like … some kind of badge of acceptance like … we support these people without really understanding them or learning about them (Lauren, ‘has only had relationships with men’, United Church of Canada, woman)

I do believe that my religion just doesn’t allow [queer identity] but for people who are gay … and are Muslim … I just feel a lot of sympathy for them … I’m trying really hard to reconcile my beliefs … and I’ve never had that before – this massive gap between the two (Jasmina, heterosexual Muslim woman, UK)

While Lauren’s religious tradition was accepting of sexual diversity, she felt this was tokenistic and naively celebratory. Meanwhile, Jasmina experienced an irreconcilability that had become difficult for her to manage, given her commitment to her religion. These accounts highlight the impact religious institutions have on heterosexuals who also desire inclusive spaces (Cuthbert and Taylor, 2018), and the challenges faced when their religious institutions fall short.

Paige, a heterosexual Roman Catholic female in Canada, expressed dismay at Catholic teachings about homosexuality. She regularly attended church, participated in the church choir, prayed daily and used Catholic teachings to inform her decision making. She strongly agreed with the statement, ‘Heterosexuality and homosexuality should be treated equally’. Her aunt is bisexual, prompting her to reflect on her attitudes in the video diary:

[When] Roman Catholic people or groups [say] that … that homosexuality is a sin or is wrong … it makes me feel so awful … who am I to judge somebody … it makes me feel so sad that if you're part of the homosexual community that you can’t also be part of your own religious upbringing … My job is to love people and to love [my] neighbour just like I love myself and to … to follow the commandments set upon us by god and to … to really just be a good person and to have a good heart … being hateful towards a huge group of people is … not part of my spirituality.

Paige utilised the Christian commandment to love one’s neighbour, as well as not judging others, resonating with Pope Francis’ 2013 comment on homosexuality: ‘Who am I to judge?’ She couched her account in religious language, supporting this with a positive emotional response, emphasising love. Evoking love is a similar strategy to those participants opposed to queer people, but Paige viewed such individuals as being ‘hateful’. Paige emphasised theological confidence in asserting that Jesus would not judge, disrupting established narratives she had encountered within Catholicism. Paige was making a powerful claim that her religious tradition contained the necessary tools to promote an inclusive and equality-bearing approach to homosexuality. Given the self-directed nature of the video diary, this focus by heterosexuals on queer issues was relatively unusual, but was foregrounded by Paige’s familial relationships.

Rashida, a heterosexual Muslim, had strongly agreed with the statement, ‘Heterosexuality should be the only expression of human sexuality’ but had also agreed that ‘Heterosexuality and homosexuality should be treated equally’. This may appear contradictory, but Rashida’s complex account was detailed in her video diary. The issue of queer identity weighted heavy on her mind for two reasons: firstly, she was a fan of EastEnders, a popular British soap which featured a storyline about a gay Muslim (Syed), dating a non-Muslim (Christian). Secondly, she was supporting a gay-identifying non-Muslim friend. Her views were refracted through the EastEnders storyline:

[W]hen I am watching [EastEnders] I never ever, as a Muslim, feel that Syed shouldn’t be gay. But in Islam it does say that homosexuality is not something that is acceptable at all and it is a vile act. But being a Muslim and being able to watch Syed and Christian together, I don’t really think it is a bad thing … It is not something perhaps to be utterly and truly honest that I would accept for a family member … [But] if [Syed] did carry on with his gay relationship I would actually be happy for him … . [My] very close friend … is gay … he hasn’t admitted it to anybody because he feels shame … I think that has made me sensitive about what gay people actually go through.

This encapsulates the contradiction that Rashida also expressed at the questionnaire stage. When she reads the issue of queer identity through the lens of her religious tradition, she sees being queer as unacceptable – even aligning it emotively with a ‘vile act’. Yet when confronted with everyday cases, she reacts differently. Meanwhile, when she thinks about the possibility of a family member being gay, this is too challenging to contemplate. But when she is thinking this through the fictional character on screen, she foregrounds his happiness (and her happiness in reacting to a ‘happy’ ending). Broader media exposure displaces a strict religious interpretation (Adamczyk, 2017). On the one hand, in her own life, Rashida can see the many challenges for queer Muslims. But this is suspended in the fictional account, where happy endings are expected.

The EastEnders storyline profoundly impacted Rashida. Despite Rashida acknowledging her own apprehension about encountering a family member who was gay, she was critical of the negative reaction Syed experienced from his family. Rashida’s case indicates the complexity of opinion formation. Her narrative can be considered inconsistent. In her everyday life, she was encountering contradictory narratives. Emotively, she felt great empathy for gay people. At the same time she used visceral language to describe how her religious tradition viewed homosexuality – as a ‘vile act’. But this contradicted with her feelings of empathy and care. Her emotional response was absolutely central to her experience and attitude formation (Moon, 2004). Rashida’s qualitative response is one of positivity rather than negativity but she is still trying to determine this in the context of a religious tradition she interprets as being opposed to queer identity.

### Changing stance

Rashida’s aforementioned account indicates the complexity surrounding attitudes, and the various entangled layers. Her account encapsulated tension, contradiction and potential movement in her attitudes. Other participants belonging to religions endorsing a conservative view also demonstrated that attitudes are not fixed and static. This occurred even when the view expressed continued to be conservatively aligned. Iqbal, a UK-based heterosexual Muslim man, said in interview:

When I was younger I was quite intolerant to homosexuals but now I have calmed down and have a few friends like that. At University you mature up. When you are kids, when you are trying to insult someone you call them “gay”. You have the mindset that if you are gay you are going to get bullied. At Uni you live with it and you can’t force anything upon them. In my religion it is not allowed, but you can’t force anything upon them.

Iqbal’s questionnaire response had failed to capture the multiple levels to his attitude formation. He admits not only his previous ‘intolerance’ to homosexuality, but implies involvement in bullying behaviour. His previous orientation was that homosexuality was wrong, should be policed and deserved to be punished. His current position was that homosexuality was ‘not allowed’, which he interpreted as being religiously conclusive. But he no longer generated negative actions through his views, and instead adopted a ‘live-and-let-live’ approach. Although this can still be deemed negative, it does represent a significant shift at the level of behaviour. Had a survey mapped his attitudes at these two points in time, they would have recorded the same response, despite much emotional diversity in the attitude conveyed.

Despite his conservatism towards homosexuality, in his video diary, Iqbal made no reference to queer issues. This was not too surprising: Participants created the video diaries independently, and it was queer^5^ participants who were more likely to reflect upon queer issues. It was rare for heterosexuals to discuss queer issues, other than fleetingly. But digging into Iqbal’s video diary revealed that his emotional investment in being opposed to homosexuality was minimal. Iqbal discussed how young Muslim men received a bad reputation in his town for gang-involvement. In a context where Muslim masculinity was problematised, Iqbal was trying to cultivate an identity as a good Muslim, to counteract broader stereotypes:

Muslim youths are giving a very bad name to Islam. Nearly all the youths are smoking weed in parks and everything … Our Prophet (peace be upon him) said that the best way to live and keep others around you is to have the best character out of everyone

Praying five times a day and attending mosque was important in enriching Iqbal’s piety. Iqbal was attempting to cultivate an identity that was distinct from fellow Muslim youth. This was the immediate framing for him. He utilised religious resources – displaying his piety through charity work, and prayer. Queer issues did not feature on his radar and anti-queer feeling was not used to bolster his masculinity (as might have previously been the case in the UK context).

Like Iqbal, Sabrina, a UK-based heterosexual Muslim woman, ticked the ‘strongly agree’ category for both conservative questionnaire statements, and confirmed this view in her interview where she said that ‘I disagree with homosexuality’. Yet she then conveyed significant attitudinal changes, precipitated by encountering queer people at work:

I got talking to them and they’re just normal people … I care about them now. But with the personal life and sexuality and having boyfriends or girlfriends that’s just very personal to them and they keep that to themselves … when we were discussing anything from art to … food and what not, that’s just normal conversation. But their personal life, that’s kept to themselves.

Interaction with queer individuals was paramount to changing Sabrina’s view. But although her orientation has shifted dramatically (from a position of intolerance to one of care), she still aligned herself with the most conservative questionnaire responses. She wanted sexuality excluded from the conversation. As Ahmed (2004: 148) notes, ‘Queer subjects may also be “asked” not to make heterosexuals feel uncomfortable by avoiding the display of signs of queer intimacy’. But the story did not end there. Sabrina further reflected on this friendship, recalling a café encounter where they discussed an attractive stranger:

[I]t came upon me that we both find him quite attractive … Just having that discussion I found that quite easy and [it] wasn’t that difficult but if it’s something they would speak about sexually and what they did, I’d find that uncomfortable.

Sabrina had relocated her boundaries; she was now able to acknowledge her queer clients’ sexuality and sexual desire. She no longer expected them to police their behaviour so that they were barred from discussing their personal life. But she still expected discussion of sexual activities to be off-limits. Therefore, Sabrina was in a position to accept queer people as friends, even at the same time that she strongly disagreed with their sexual identity.

## Discussion and conclusion

This article has analysed attitudes to queer identity in similarly constituted contexts where queer equality is endorsed – Canada and the UK – using data collection techniques that mirrored each other. Younger people are typically more positive toward queer identity, yet those identifying religiously are more likely to be negative, making heterosexual young religious adults an important group to study, as they manage both religious and secular youth discourses on sexuality. Both Christianity and Islam have been understood as taking a conservative stance on queer identities, although there are numerous examples within these religions of more liberal interpretations (Yip, 2012). As Murray (2009) argues, it is often easy to cast whole communities, or even religious groups, as being homophobic, but often the story is more nuanced. While the Canadian data revealed more liberal religious attitudes to sexuality, there were important parallels between the studies on how views were rationalised. This article indicates that much is missed when a quantitative approach is relied upon as an accurate measure of attitudes. The data show that attitude formation is a complex endeavour that entails change, fluidity, and inconsistency, emphasising the importance of utilising qualitative methods, with questionnaires often being blunt instruments (Day and Lee, 2014; Rosik et al., 2007). The qualitative approaches not only revealed complexity regarding how opinion is verbalised and affirmed in both contexts, but also highlighted how attitudes are also emotionally created and embodied (Blumer, 1936).

Those who firmly disagreed with queer identities often utilised scientific narratives to support their stance. This fulfilled various purposes. It disassociated them from the label ‘homophobic’, given that ‘homophobia’ is associated with the negative emotion of fear. By evoking the rational, it endorses the claim that one is not governed by one’s emotions. It also distanced their views from an explicitly religious one. Asserting non-religious reasoning enabled those opposed to queer identities to decouple their arguments from the ‘baggage’ of religion, given the way in which ‘religion’ is often associated with homophobia. But this fails when it is understood that a rationalising and scientific outlook is itself an emotional register (Ahmed, 2004). Seeking out the rational does not work as a means of avoiding emotional responses in attitude formation.

As both Canada and the UK are perceived as being liberal toward queer identities (Nash and Browne, 2015), those opposed to queer identities knew that their perspective was out of sync with broader attitudes. Given the way in which a scientific approach has been erroneously understood as overcoming emotion, this created a problem for participants, in that they did not want to be construed as unfeeling. They wanted to demonstrate good personhood – their ability to feel and have compassion. This was usually achieved by evoking the emotion of ‘love’ – with many attesting that although they were opposed to homosexuality, they loved all, irrespective of sexual orientation. Love evokes notions of the exemplary self (Ahmed, 2004). But heterosexuality is projected onto queer people as the ideal state, and as the ‘best’ way to live. The strength of this vision means that others are only loved to the extent that they can commit to and fulfil these heteronormative ideals. The ‘problem’, as it is imagined by those opposed to queer people, is displaced onto the bodies of non-heterosexuals. To belong in this community of ‘love’ requires non-heterosexuals to contort their bodies to fit in; to forgo desire and live only on the terms dictated by compulsory heterosexuality and heteronormativity.

Others were far more conflicted and agonised over how to navigate a terrain where participants interpreted their religious tradition as being fundamentally opposed to homosexuality, yet witnessing the harms caused. Relationality had a large role to play; various studies have emphasised how connections with queer individuals engender positive attitudes (Adamczyk, 2017; DellaPosta, 2018). Those participants who personally knew someone facing the challenges of being queer displayed clear compassion. Unlike the previous group, these participants had a more expansive understanding of love, that tried (often over time) to accept the person for who they were, without condition. Yet even here, conflicts emerged, as some found certain issues (e.g. types of intimate sexual practice) insurmountable.

Meanwhile, those heterosexuals supporting an equality position did one of two things: they either critiqued how non-heterosexuals were treated in their religious communities, or they articulated that their religious tradition was supportive and welcoming. In neither case did this facilitate broader activism. If their religious tradition was considered welcoming, then heterosexual participants could vicariously benefit from this without further action needed. If religions were found wanting in their support for non-heterosexuals, this could be a subject of complaint but did not prompt activism. The differences, however, were at the interpersonal level. Those who were supporting a queer person reflected upon the injustices experienced, as they tried to help. But this was rarely about angling for structural change.

Heterosexuals said little about queer issues in the context of their day-to-day lives. Even those who had professed a strong oppositional attitude were not preoccupied with this issue. This gives pause for thought regarding why there is a consensus that religious people *will* have an opinion on queer identities. This may say more about the broader discourses regarding the presumed relationship between sexuality and religion (Puar, 2007), than the actual concerns of specific religious individuals. Indeed, in other locations, religious reasoning has been utilised to incite violence against queer people, especially when there is a feeling that queer people threaten the conceptualisation of nationhood. For example, in Indonesia, Boellstorff (2009) charts the increased violence meted out to queer people by groups representing political Islam, linking this to challenges to Indonesian masculinity at the national level. In the Canadian and British contexts, very rarely were participants motivated to be active in their opposition to queer people, whether through their religious institution or beyond it (see Nash and Browne, 2015 for more details of organisations in the Canadian and British context – both secular and religious – which actively resist queer equalities). Participants may have had negative feelings, but those feelings did not lead to violence or abuse. Given the way nationhood in both contexts is tied to strong narratives of sexuality equality and rights for queer people, more often than not, participants opposed were trying to articulate their good personhood in spite of their negative attitudes. Whatever their perspective, heterosexual young adults in our studies were little-motivated to get involved in any activism around sexuality. A negative view to queer people did little to bolster their nationhood credentials.

We recognise the limitations of these studies. Both projects mapped attitudes at a moment in time and did not measure longitudinal shifts. We also recognise that some of the questionnaire questions were framed solely around homosexuality; if lesbianism had been more prominent in the ways the questions were framed, the results may have differed (Herek, 2004). What we hope to have achieved is a widening of the types of methods considered for mapping attitudes, and a questioning of the causal links often assumed between religious affiliation and an individual’s attitude. It is important to examine why religion is considered the independent variable, and what a contextual focus might reveal regarding the extent to which attitudes to certain issues are salient, and the extent to which these attitudes are embodied as well as cognitive.

## Notes

1 The Canadian project explicitly modelled their study on the UK project.

2 This included the categories, nonreligious, spiritual-but-not-religious and ‘other’ identifications.

3 Participants could select more than one religious identity category.

4 The total numbers of valid cases are 497, 495 and 497, respectively. The total numbers of valid cases are 401, 399 and 401, respectively.

5 This includes a variety of identities such as lesbian, gay, bisexual, trans, queer and intersex.

6 In the Canadian study, Sikh respondents did not answer any of these questions.

## Case list

*Egan v Canada*, [1995] 2 SCR 513

*Vriend v Alberta* [1998] 1 S.C.R. 493

*Halpern v Canada* (AG), [2003] O.J. No. 2268

*Reference Re Same-Sex Marriage* [2004] 3 S.C.R. 698, 2004 SCC 79

## References

[bibr1-1363460721995756] Adam B Maticka-Tyndale E (2011) Emerging directions in sociological research on sexuality. Canadian Review of Sociology 48(3): 217–220. 22214040 10.1111/j.1755-618x.2011.01263.x

[bibr2-1363460721995756] Adamczyk A (2017) Cross-National Public Opinion about Homosexuality: Examining Attitudes Across the Globe. Oakland: University of California Press.

[bibr3-1363460721995756] Ahmed S (2004) The Cultural Politics of Emotion. Edinburgh: Edinburgh University Press.

[bibr4-1363460721995756] Blumer H (1936) Social attitudes and nonsymbolic interaction. The Journal of Educational Sociology 9(9): 515–523.

[bibr5-1363460721995756] Boellstorff T (2009) The emergence of political homophobia in Indonesia masculinity and national belonging. In Murray DAB (ed) Homophobias: Lust and Loathing across Time and Space. Durham: Duke University Press, pp. 123–145.

[bibr6-1363460721995756] Clements B (2015) Religion and Public Opinion in Britain. New York: Palgrave Macmillan.

[bibr7-1363460721995756] Corriveau P (2011) Judging Homosexuals. Vancouver: UBC Press.

[bibr8-1363460721995756] Cuthbert K Taylor Y (2018) Queer liveability: Inclusive church-scenes. Sexualities 2(5–6): 951–968.

[bibr9-1363460721995756] Day A Lee L (2014) Making sense of surveys and censuses: Issues in religious self-identification. Religion 44(3): 345–356.

[bibr10-1363460721995756] DellaPosta D (2018) Gay acquaintanceship and attitudes toward homosexuality: A conservative test. Socius 4(1): 1–12.

[bibr11-1363460721995756] Fone B (2000) Homophobia. New York: Picador.

[bibr12-1363460721995756] Foucault M (1978) The History of Sexuality. New York: Pantheon Books.

[bibr13-1363460721995756] Herek GM (2004) Beyond ‘homophobia’: Thinking about sexual prejudice and stigma in the twenty-first century. Sexuality Research and Social Policy 1(2): 6–24.

[bibr14-1363460721995756] Hooghe M Meeusen C (2013) Is same-sex marriage legislation related to attitudes toward homosexuality? Sexuality Research and Social Policy 10(4): 258–268.

[bibr15-1363460721995756] Jackson S (2006) Gender, sexuality and heterosexuality: The complexity (and limits) of heteronormativity. Feminist Theory 7(1): 105–121.

[bibr16-1363460721995756] Jakobsen JR Pellegrini A (2003) Love the Sin. Boston: Beacon Press.

[bibr17-1363460721995756] Kulick D (2009) Can there be an anthropology of homophobia? In: Murray DAB (ed) Homophobias: Lust and Loathing across Time and Space. Durham: Duke University Press, pp. 19–33.

[bibr18-1363460721995756] Maticka-Tyndale E (2008) Sexuality and sexual health of Canadian adolescents: Yesterday, today and tomorrow. Canadian Journal of Human Sexuality 17(3): 85–95.

[bibr19-1363460721995756] Midgley M (2004) The Myths We Live By. London: Routledge.

[bibr20-1363460721995756] Moon D (2004) God, Sex, and Politics. Chicago: University of Chicago Press.

[bibr21-1363460721995756] Murray DAB (2009) Introduction. In: Murray DAB (ed) Homophobias: Lust and Loathing across Time and Space. Durham: Duke University Press, pp.1–15.

[bibr22-1363460721995756] Nash CJ Brown K (2015) Best for society? Transnational opposition to sexual and gender equalities in Canada and Great Britain. Gender, Place & Culture 22(4): 561–577.

[bibr23-1363460721995756] Page S (2017) Exploring young adults’ faith lives through video diaries. In: Slee N Porter F Phillips A (eds) Researching Female Faith. London: Routledge, pp. 98–112.

[bibr24-1363460721995756] Page S Shipley H (2020) Religion and Sexualities. Abingdon: Routledge.

[bibr25-1363460721995756] Petro AM (2015) After the Wrath of God. Oxford: Oxford University Press.

[bibr26-1363460721995756] Puar JK (2007) Terrorist Assemblages. Durham: Duke University Press.

[bibr27-1363460721995756] Rhys-Taylor A (2013) Disgust and distinction: The case of the jellied eel. The Sociological Review 61: 227–246.

[bibr28-1363460721995756] Richardson D (2018) Sexuality and Citizenship. Cambridge: Polity Press.

[bibr29-1363460721995756] Roggemans L Spruyt B Van Droogenbroeck F , et al. (2015) Religion and negative attitudes towards homosexuals: An analysis of urban young people and their attitudes towards homosexuality. Young 23(3): 254–278.

[bibr30-1363460721995756] Rosik CH Griffith LK Cruz Z (2007) Homophobia and conservative religion: Toward a more nuanced understanding. American Journal of Orthopsychiatry 77(1): 10–19. 17352580 10.1037/0002-9432.77.1.10

[bibr31-1363460721995756] Ross A Sacker A (2010) Understanding the dynamics of attitude change. In: Park A Curtice J Thomson K , et al. (eds) British Social Attitudes. London: Sage, pp. 115–133.

[bibr32-1363460721995756] Siraj A (2009) The construction of the homosexual ‘other’ by British Muslim heterosexuals. Contemporary Islam 3: 41–57.

[bibr33-1363460721995756] Spencer L Pahl R (2006) Rethinking Friendship. Princeton, NJ: Princeton University Press.

[bibr34-1363460721995756] Tyler I (2013) Revolting Subjects. London: Zed Books.

[bibr35-1363460721995756] Voas D (2013) Towards a sociology of attitudes. Sociological Research Online 19: 12.

[bibr36-1363460721995756] Weeks J (2007) The World We Have Won. Abingdon: Routledge.

[bibr37-1363460721995756] Wilcox MM (2018) Queer Nuns. New York: New York University Press.

[bibr38-1363460721995756] Woodhead L (2013) Religion and Personal Life. London: Darton, Longman and Todd.

[bibr39-1363460721995756] Yip AKT (2012) Homophobia and ethnic minority communities in the United Kingdom. In: Trappolin L Gasparini A Wintemute R (eds) Confronting Homophobia in Europe. Oxford: Hart, pp.107–130.

[bibr40-1363460721995756] Yip AKT Page S (2013) Religious and Sexual Identities. Farnham: Ashgate.

[bibr41-1363460721995756] Young PD (2012) Religion, Sex and Politics. Winnipeg: Fernwood Publishing.

[bibr42-1363460721995756] Young PD Shipley H (2020) Identities under Construction. Montreal: McGill-Queen’s University Press.

